# Methionine sulfoxide reductase B3 requires resolving cysteine residues for full activity and can act as a stereospecific methionine oxidase

**DOI:** 10.1042/BCJ20170929

**Published:** 2018-02-28

**Authors:** Zhenbo Cao, Lorna Mitchell, Oliver Hsia, Miriam Scarpa, Stuart T. Caldwell, Arina D. Alfred, Alexandra Gennaris, Jean-François Collet, Richard C. Hartley, Neil J. Bulleid

**Affiliations:** 1 Institute of Molecular, Cellular and Systems Biology, CMVLS, University of Glasgow, Davidson Building, Glasgow G12 8QQ, U.K.; 2 WestCHEM School of Chemistry, University of Glasgow, Glasgow G12 8QQ, U.K.; 3 de Duve Institute, Université catholique de Louvain, Avenue Hippocrate 75, 1200 Brussels, Belgium; 4 WELBIO, Avenue Hippocrate 75, 1200 Brussels, Belgium

**Keywords:** methionine sulfoxide reductase, endoplasmic reticulum, post-translational modification, oxidation–reduction

## Abstract

The oxidation of methionine residues in proteins occurs during oxidative stress and can lead to an alteration in protein function. The enzyme methionine sulfoxide reductase (Msr) reverses this modification. Here, we characterise the mammalian enzyme Msr B3. There are two splice variants of this enzyme that differ only in their N-terminal signal sequence, which directs the protein to either the endoplasmic reticulum (ER) or mitochondria. We demonstrate here that the enzyme can complement a bacterial strain, which is dependent on methionine sulfoxide reduction for growth, that the purified recombinant protein is enzymatically active showing stereospecificity towards *R*-methionine sulfoxide, and identify the active site and two resolving cysteine residues. The enzyme is efficiently recycled by thioredoxin only in the presence of both resolving cysteine residues. These results show that for this isoform of Msrs, the reduction cycle most likely proceeds through a three-step process. This involves an initial sulfenylation of the active site thiol followed by the formation of an intrachain disulfide with a resolving thiol group and completed by the reduction of this disulfide by a thioredoxin-like protein to regenerate the active site thiol. Interestingly, the enzyme can also act as an oxidase catalysing the stereospecific formation of *R*-methionine sulfoxide. This result has important implications for the role of this enzyme in the reversible modification of ER and mitochondrial proteins.

## Introduction

Both cysteine and methionine residues in proteins are susceptible to oxidation by reactive oxygen species [1]. This oxidation can lead to an alteration of protein function or to a signalling event resulting in a change in gene expression [2]. The cell has many pathways that can reverse these side chain modifications that involve either the glutathione or thioredoxin pathways [3,4]. These pathways can act directly to reduce modified thiols or can act indirectly through enzymes called methionine sulfoxide reductases (Msr) to reduce modified methionine [5].

In mammalian cells, there are up to four known isoforms and two splice variants of the enzyme that differ in their cellular location and their stereospecificity [5]. Since the non-enzymatic oxidation of methionine by ROS results in the formation of a diastereomeric mixture of methionine *S*-sulfoxide and methionine *R*-sulfoxide [6], two enzymes are required to catalyse complete reduction. The MsrA enzymes specifically reduce the *S*-epimer, whereas the MsrB enzymes reduce the *R*-epimer [7]. There are two main splice variants of MsrA in mammalian cells that differ at their N-terminal region: one having a mitochondrial targeting signal and localising to this organelle and the other being localised to the cytosol and the nucleus [8]. There are three known MsrB enzymes. MsrB1 is localised to the cytosol and is the only isoform that contains a selenocysteine at its active site. MsrB2 is localised to mitochondria, whereas MsrB3 exists as two main splice variants A and B, containing either a signal sequence targeting the protein to the ER or to the mitochondria, respectively [9]. Cleavage of either signal sequence results in an identical mature protein. As there is only one Msr known to be localised to the ER, MsrB3A, its stereospecificity suggests that only half the non-enzymatically oxidised methionine residues in proteins present in the ER are reduced or that there is an epimerase capable of converting the *S-* into the *R-*epimer. In contrast with MsrA, all the MsrB enzymes are zinc-binding proteins. The zinc centre seems to play a structural rather than catalytic role [10].

Previous work on purified recombinant MsrB3 has revealed its ability to reduce methionine sulfoxide with apparent specificity for the *R*-epimer [9–11]. The first step in the Msr reaction is the formation of a sulfenic acid intermediate on the catalytic Cys of the enzyme and the concomitant release of reduced methionine. Characterisation of the role of individual cysteines in the enzymatic process for MsrB3 has suggested that the mechanism of recycling involves the direct reduction of this active site thiol by a thioredoxin domain-containing protein [11]. This contrasts with MsrB1 which acts via a two-step process involving an internal resolving cysteine that reduces the sulfenylated active site thiol forming a disulfide which is reduced further by thioredoxin [10], but is consistent with the proposed mechanism for MsrB2 which also does not involve a resolving cysteine [12].

Most of the MsrA enzymes have a single resolving cysteine and recycle the active site via a two-step mechanism. However, a subclass of MsrA enzymes have two resolving cysteines which are thought to act via a three-step process involving disulfide exchange which results in the formation of an internal disulfide bond between the resolving cysteines that is reduced by thioredoxin or low molecular mass thiols [13–15]. This particular subclass of MsrA enzymes acts as a stereospecific reductase, but has also been shown to catalyse the stereospecific oxidation of methionine, a reaction that does not require thioredoxin and results in exclusively the *S*-epimer [16,17].

The functional role of MsrB3 in cells is poorly defined. The poor growth on methionine *R*-sulfoxide phenotype demonstrated by a yeast strain carrying both MsrA and MsrB deletions was complemented by mouse MsrB3, but only when this protein was targeted to the mitochondria [9]. This suggests that when mouse MsrB3 is expressed in the yeast ER, it is either non-functional or unable to reduce free methionine sulfoxide for further metabolism. However, depletion of the ER-localised MsrB3A in mammalian cells leads to ER stress, whereas overexpression of MsrB3A is protective [18]. These results suggest an important role for MsrB3A in preventing the accumulation of misfolded proteins. Functionally null mutants of the MsrB3 gene also lead to deafness, though whether this is caused by lack of ER or mitochondrial function is unclear [19,20].

To provide further insight into the function and enzymatic mechanism of MsrB3A, we characterised the properties of the recombinant protein against a model substrate using different electron donors. By carrying out selective mutagenesis of cysteine residues, we show that the enzyme is most efficiently recycled by a three-step process requiring two potential resolving cysteine residues. These results contrast with previous interpretations and suggest that resolving cysteine(s) are required for mammalian MsrB1 and MsrB3. In addition, we demonstrate that MsrB3 is able to act as an oxidase suggesting a role for methionine oxidation in the regulation of ER protein function.

## Experimental procedures

### Reagents and antibodies

Most reagents were purchased from Sigma–Aldrich (Dorset, U.K.), with some enzymes from Promega (Southampton, U.K.) unless otherwise stated. Rabbit monoclonal anti-MsrB3 antibody was purchased from Abcam (Cambridge, U.K.) and mouse anti-His antibody was purchased from Proteintech Europe (Manchester U.K.). Human thioredoxin (trx) and thioredoxin reductase (trxR) used in enzyme assays were purchased from IMCO (Stockholm, Sweden).

### Synthesis of dabsylated methionine and dabsylated methionine sulfoxides

Adapting a previous procedure [21], *N-*dabsyl-l-methionine was synthesised as follows: 4-(4-dimethylaminophenylazo)benzenesulfonyl chloride (dabsyl chloride) (100 mg, 0.31 mmol, 1.0 eq) was added to a solution of l-methionine (55 mg, 0.37 mmol, 1.2 eq) and sodium bicarbonate (156 mg, 0.85 mmol, 6.0 eq) in acetone (40 ml) and water (5 ml). The solution was stirred at RT for 2 h and then the acetone was removed under vacuum. The solution was slowly acidified and the resulting precipitate filtered off and washed with water. The solid was purified by automated chromatography on a Biotage Isolera using a 10 g SNAP ultra-cartridge and eluting with CH_2_Cl_2 _: MeOH (100 : 0 to 91 : 9) to give the *N-*dabsyl-l-methionine as an orange solid. ^1^H NMR (400 MHz: CDCl_3_) *δ*_H_: 1.80–1.89 (1H, m, CHC*H_A_*H_B_), 1.99–2.06 (1H, m, CHCH_A_*H_B_*), 2.02 (3H, s, SCH_3_), 2.44–2.58 (2H, m, SCH_2_), 3.10 (6H, s, NCH_3_), 4.08–4.13 (1H, m, CH), 5.57 (1H, d, *J* = 9.1 Hz, NH), 6.73 (2H, d, *J* = 9.3 Hz, ArH), 7.83–7.91 (6H, m, ArH). A 1 : 1 mixture of *N-*dabsyl-l-methionine *R*-sulfoxide and *N-*dabsyl-l-methionine *S*-sulfoxide was synthesised using a similar procedure, but with an epimeric mixture of l-methionine *R*-sulfoxide and l-methionine *S*-sulfoxide as the starting material and using a gradient of CH_2_Cl_2 _: MeOH (100 : 0 to 75 : 25) in the purification. ^1^H NMR (400 MHz: CD_3_OD) *δ*_H_: 2.00–2.10 (1H*^R^*^&*S*^, m, CHC*H_A_*H_B_), 2.24–2.31 (1H*^R^*^&*S*^, m, CHCH_A_*H_B_*), 2.63 [3H*^R^* ^or*S*^, s, S(O)CH_3_], 2.64 [3H*^R^* ^or *S*^, s, S(O)CH_3_], 2.79–2.89 [1H*^R^*^&*S*^, m, S(O)C*H_A_*H_B_], 2.92–3.02 [1H*^R^*^&*S*^, m, S(O)CH_A_*H_B_*], 3.14 (6H*^R^*^&*S*^, s, NCH_3_), 4.08 (1H*^R^*^&*S*^, dd, *J* = 9.1, 4.6 Hz, C*H*NH), 6.87 (2H*^R^*^&*S*^, d, *J* = 9.2 Hz, ArH), 7.89 (2H*^R^*^&*S*^, d, *J* = 9.1 Hz, ArH), 7.90–7.94 (2H*^R^*^&*S*^, m, ArH), 7.97–8.00 (2H*^R^*^&*S*^, m, ArH).

### DNA constructs

Human MsrB3 DNA constructs, lacking any N-terminal signal sequence, containing a GST tag at C-terminus were synthesised by GenScript (New Jersey, U.S.A.). Mutagenesis was performed as described previously [22] using GST-tagged MsrB3A as a template.

### MsrB3A complementation assay

Human MsrB3, lacking any signal sequence, was cloned into pAG177 vector, which allows expression from the *tac* promoter. The construct was transformed into *Escherichia coli* strain JB590, a methionine auxotroph, which lacks the genes encoding MsrA, MsrB, MsrC and BisC, and therefore is not able to use methionine sulfoxide as the only source of methionine [23]. This transformed strain and the JB590 strain containing an empty pAG177 vector were grown on M63 medium agar plates containing 20 µg/ml Met-O (mixture of both epimers), 10 µM IPTG and 200 µg/ml ampicillin for 48 h at 37°C.

### Preparation of purified proteins

All MsrB3 proteins were expressed and purified using *E. coli* BL21-DE3. GST-MsrB3 was expressed for 16 h at 18°C in the presence of 0.2 mM IPTG. Cells were disrupted by two passes through a chilled French pressure cell at 12 000 psi. The resulting crude extract was centrifuged for 45 min at 30 000×***g*** and 4°C. Cells were lysed and GST-MsrB3 bound to GSH-Sepharose (GE Healthcare, Buckinghamshire, U.K.). MsrB3 was eluted by on-column cleavage with precision protease (GE Healthcare) in accordance with the manufacturer's instructions. Proteins were reduced by incubation with 5 mM DTT for 15 min at room temperature, then DTT removed by a Superdex 200 gel filtration column (GE Healthcare). The purification of His-tagged trx and its mutants has been described previously [24]. Each protein was quantified using the relevant 280 nm absorption extinction coefficient.

MsrA from *E. coli* containing a C-terminal His-tag was expressed and purified as described previously [25] with some modifications. Briefly, BL21 (DE3) cells harbouring the pET21MsrA vector were grown at 37°C and induced with 1 mM IPTG for 3 h. Cells were then pelleted, resuspended in buffer A (50 mM NaPi, pH 8.0, containing 300 mM NaCl) and disrupted by two passes through a chilled French pressure cell at 12 000 psi. The resulting crude extract was centrifuged for 45 min at 30 000×***g*** and 4°C. The supernatant was loaded on a 5 ml Histrap FF column (GE Healthcare) previously equilibrated with buffer A and was eluted by applying a linear gradient of imidazole (from 0 to 300 mM) in buffer A. Selected elution fractions containing MsrA-His were pooled, concentrated using a 5 kDa cut-off Vivaspin 15 (Sartorius) device and buffer-exchanged on a PD-10 column (GE Healthcare) equilibrated with 50 mM NaPi (pH 8.0) and 150 mM NaCl.

### Assay for MsrB3 reductase activity

The reaction mixture (200 µl) contained 20 mM Tris–HCl buffer, pH 7.5, containing 150 mM NaCl, 500 µM dabsyl-methionine sulfoxide, 20 µM MsrB3A protein and 10 mM DTT or a trx recycling system (5 µM trx, 65 nM trxR and 5 mM NADPH). The reaction was carried out at 37°C for various lengths of time before being loaded onto a C18 column (25 cm Apex ODS 5 µ, Jones Chromatography) equilibrated in 100 mM sodium acetate (pH 6) and 30% acetonitrile. An Akta Micro-Chromatography system was used controlled by the Unicorn software (v.5.2). The sample was eluted using a linear gradient (from 30 to 70% over three column volumes) of acetonitrile and the dabsyl derivatives were monitored by absorbance at 460 nm. The fraction of reduced product was calculated by dividing the amount of dabsylated methionine produced by the total amount of dabsyl-methionine *R*-sulfoxide and dabsyl-methionine at each time point.

### Assay for MsrB3 oxidase activity

Wild-type or mutant MsrB3 was first oxidised by incubation with a 20-fold molar excess of methionine sulfoxide for 30 min at 37°C. The excess methionine sulfoxide was then removed by passing through a desalting spin column twice. Oxidised MsrB3 (250 µM) was then incubated with an equimolar concentration of dabsyl-methionine for 12 h at 37°C. Product formation was then evaluated by RP-HPLC as described above.

### Immunoblotting

Samples containing 20 mM Tris–HCl buffer (pH 7.5), 150 mM NaCl, 500 µM methionine sulfoxide, 10 µM MsrB3 and/or trx proteins were incubated at 37°C for 1 h before being separated on a 15% SDS–PAGE gel and then proteins being transferred to a nitrocellulose membrane (GE Healthcare). Non-specific binding was blocked using 5% (w/v) non-fat dried skimmed milk in Tween/Tris-buffered saline (TTBS) [50 mM Tris–HCl buffer, pH 7.5, containing 150 mM NaCl and 0.1% (v/v) Tween 20]. The same membrane was incubated with both primary antibodies for 16 h at 4°C in TTBS. The fluorescent secondary antibodies were diluted 1 : 5000 in TTBS and incubation was performed in a light-shielded box for 45 min at room temperature. For Msr3B antibody, the anti-rabbit secondary antibody was conjugated to a probe that emits at 700 nm (red), whereas for trx the anti-mouse secondary is conjugated to a probe that emits at 800 nm (green). Specific proteins were visualised using an Odyssey LI-COR Sa imaging system on two independent channels at 700 and 800 nm.

## Results

### Expression and characterisation of human MsrB3

**Figure 1.**
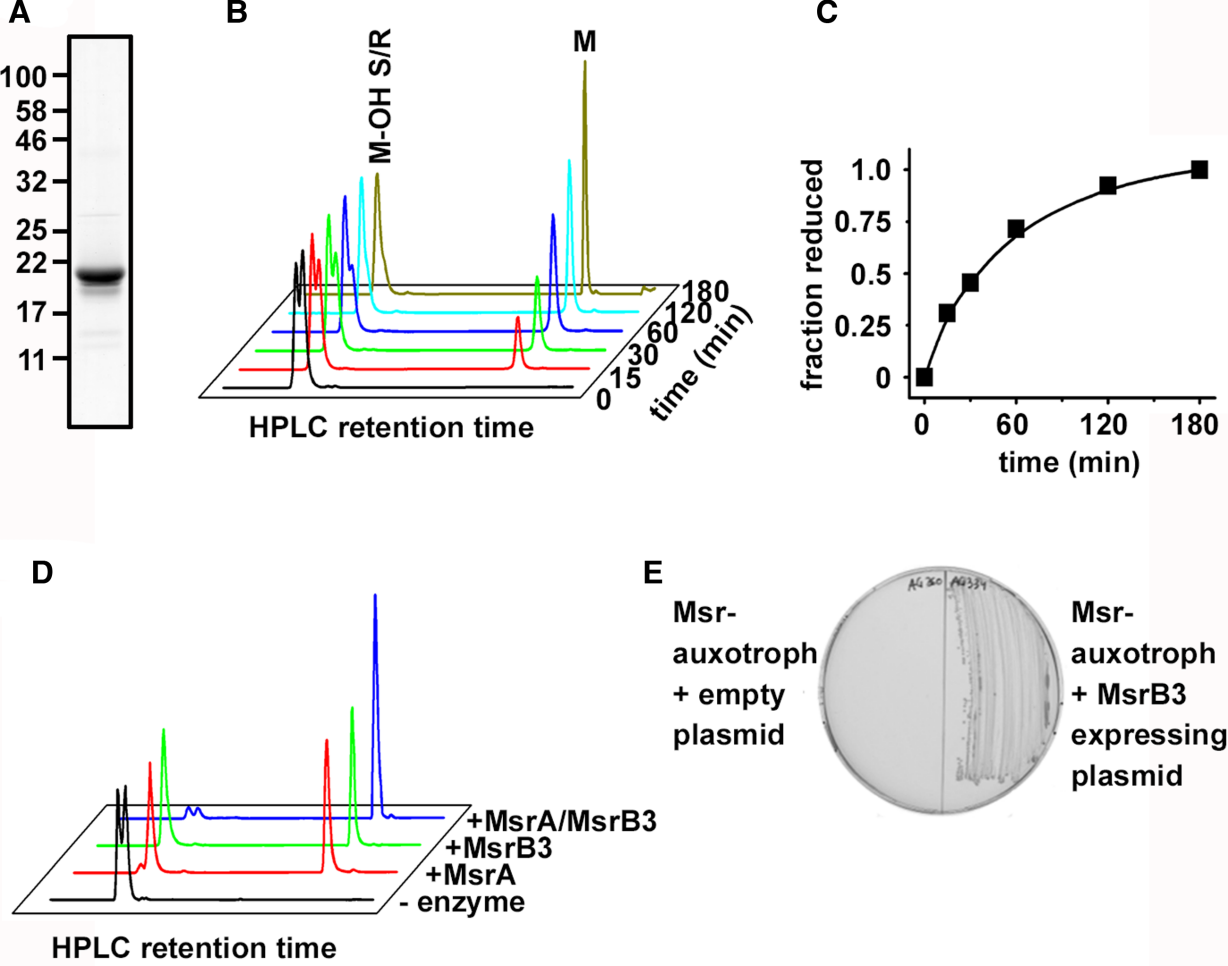
Characterisation of the reductase activity of MsrB3. (**A**) Partially purified MsrB3 after expression in *E. coli* was separated by SDS–PAGE before Coomassie blue staining. (**B**) Activity of MsrB3 was assayed in the presence of a mixture of methionine *R*- and *S*-methionine sulfoxide and DTT. Incubation was carried out for the times indicated prior to separation of the product and reactant by RP-HPLC using a linear gradient from 30% to 70% acetonitrile. Dabsyl-methionine (M) (retention time ∼38.5 min) was separated from methionine sulfoxide (M-OH S/R) (retention time between 24 and 25 min). Dabsylated molecules were detected by following absorption at 460 nm. The same experiment was carried out at least three times with similar results. (**C**) Quantification of the time course carried out in (**B**) with the fraction reduced plotted against time. (**D**) RP-HPLC of reactions carried out in the presence of dabsyl-methionine sulfoxide and DTT in the absence (black) or presence of MsrA (red), MsrB3 (green) or MsrA with MsrB3 (blue). (**E**) A bacterial strain auxotrophic for methionine was transfected with empty plasmid (left) or MsrB3-expressing plasmid (right) prior to plating on an agar plate containing methionine sulfoxide as the sole source of methionine.

To evaluate the enzymatic activity of MsrB3, we first prepared recombinant mature protein lacking any signal sequence. The protein was expressed in *E. coli* as a fusion protein with GST which was subsequently cleaved after purification to yield the 21 kDa MsrB3 protein (Figure 1A). The addition of a mixture of isomers of dabsylated methionine sulfoxide to the enzyme in the presence of DTT led to the production of methionine (Figure 1B,C). The product was separated from the reactant by RP-HPLC, which also separated the substrate into two peaks presumably representing the *R-* and *S-*epimers. Only the second of these two peaks was reduced by MsrB3 demonstrating stereospecificity in its enzymatic reaction. To determine which isomer was reduced by MsrB3, we purified *E. coli* MsrA which is known to reduce methionine *S*-sulfoxide. We then carried our enzymatic assay with MsrA or B3 alone or together (Figure 1D). MsrA reduced the first but not the second substrate peak, whereas the combination of both enzymes reduced both peaks. These results demonstrate that MsrB3 is enzymatically active towards methionine sulfoxide and specifically reduces the *R*-epimer.

We evaluated whether MsrB3 was active *in vivo* by determining its ability to complement an *E. coli* strain that is depleted of all endogenous Msrs and is auxotrophic for methionine. We have previously shown that when methionine sulfoxide is provided as a mixture of isomers in the medium, this strain is unable to grow as it cannot convert methionine sulfoxide into methionine. When human MsrB3 was expressed in this strain, it was now able to grow on medium containing methionine sulfoxide. These results demonstrate that MsrB3 is able to function *in vivo* to complement a bacterial strain deficient in endogenous Msr.

### Defining the catalytically important thiols

**Figure 2.**
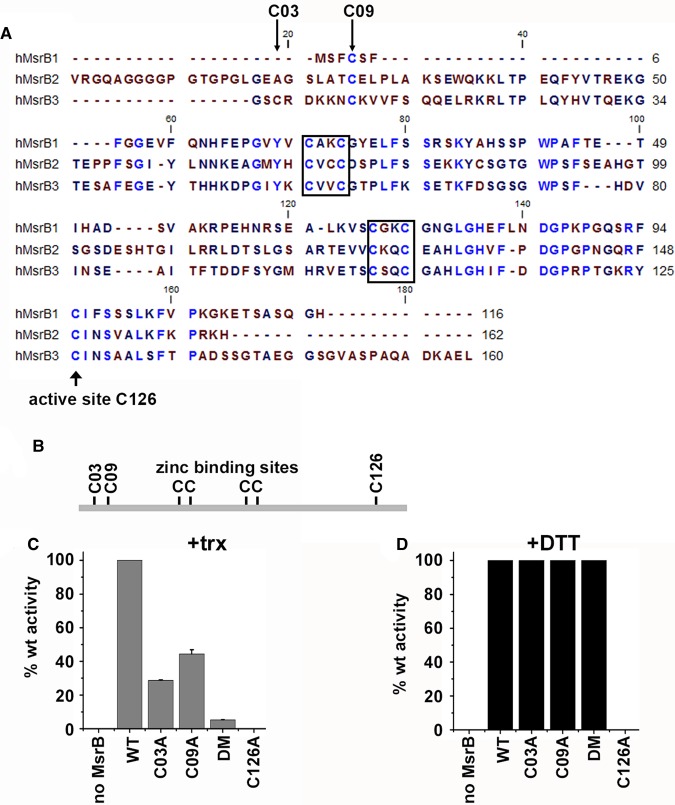
Identification of the active site and resolving cysteine residues in MsrB3. (**A**) Alignment plot of the human MsrB family of proteins. Identical conserved residues are in blue. Boxes depict two CXXC motifs that are thought to be involved in zinc binding. The putative active site cysteine is indicated with an arrow below the sequence. (**B**) Schematic of MsrB3 illustrating the positions of the cysteine residues. (**C** and **D**) Msr3B activity assays carried out with either thioredoxin (trx) or DTT as an electron donor. Assays were carried out for 120 min in the absence or presence of MsrB3 wild-type or cysteine mutants as indicated. DM: C03A, C09A double mutant. Error bars represent ± SD from three independent experiments.

An alignment plot of the three human MsrB proteins illustrates that there are many highly conserved cysteines (Figure 2A,B). These include the putative active site thiol (C126 — MsrB3 mature sequence numbering) and two CXXC motifs that constitute a zinc-binding site. The resolving cysteine in MsrB1 is located within the N-terminal region. While this cysteine residue is conserved in MsrB2 and MsrB3 (C09), it is not thought to carry out a role as a resolving cysteine in these enzymes [11]. MsrB3 contains an additional non-conserved cysteine (C03) close to its N-terminus which has the potential to act as a resolving cysteine. To evaluate a potential role for C03 and C09 in resolving the active site sulfenylated thiol, we mutated these residues to alanine individually or together and determined the effect on enzymatic activity using DTT or thioredoxin as electron donors (Figure 2C,D). We also verified that C126 is the active site thiol as mutating this residue to alanine resulted in no enzymatic activity. Single mutation of C03 or C09 to alanine resulted in a dramatic reduction of activity when thioredoxin was used as an electron donor. When both residues were mutated, almost no activity was recorded indicating that one or other of these two cysteines is required for optimal activity in the presence of thioredoxin. Wild-type level of activity for the single and double C03 and C09 mutants was recorded when DTT was used as the electron donor, indicating that these mutations did not effect the ability of the enzyme to catalyse methionine sulfoxide reduction suggesting that they still folded correctly. Taken together, the results with these mutants strongly suggest that either C03 or C09 can act as a resolving thiol and that thioredoxin can only directly reduce the sulfenylated active site thiol inefficiently. For maximal activity with thioredoxin, both C03 and C09 need to be present, suggesting that the preferred mechanism is via an internal disulfide between these two cysteines.

### Direct reduction of MsrB3 by thioredoxin mediated by resolving cysteines

**Figure 3.**
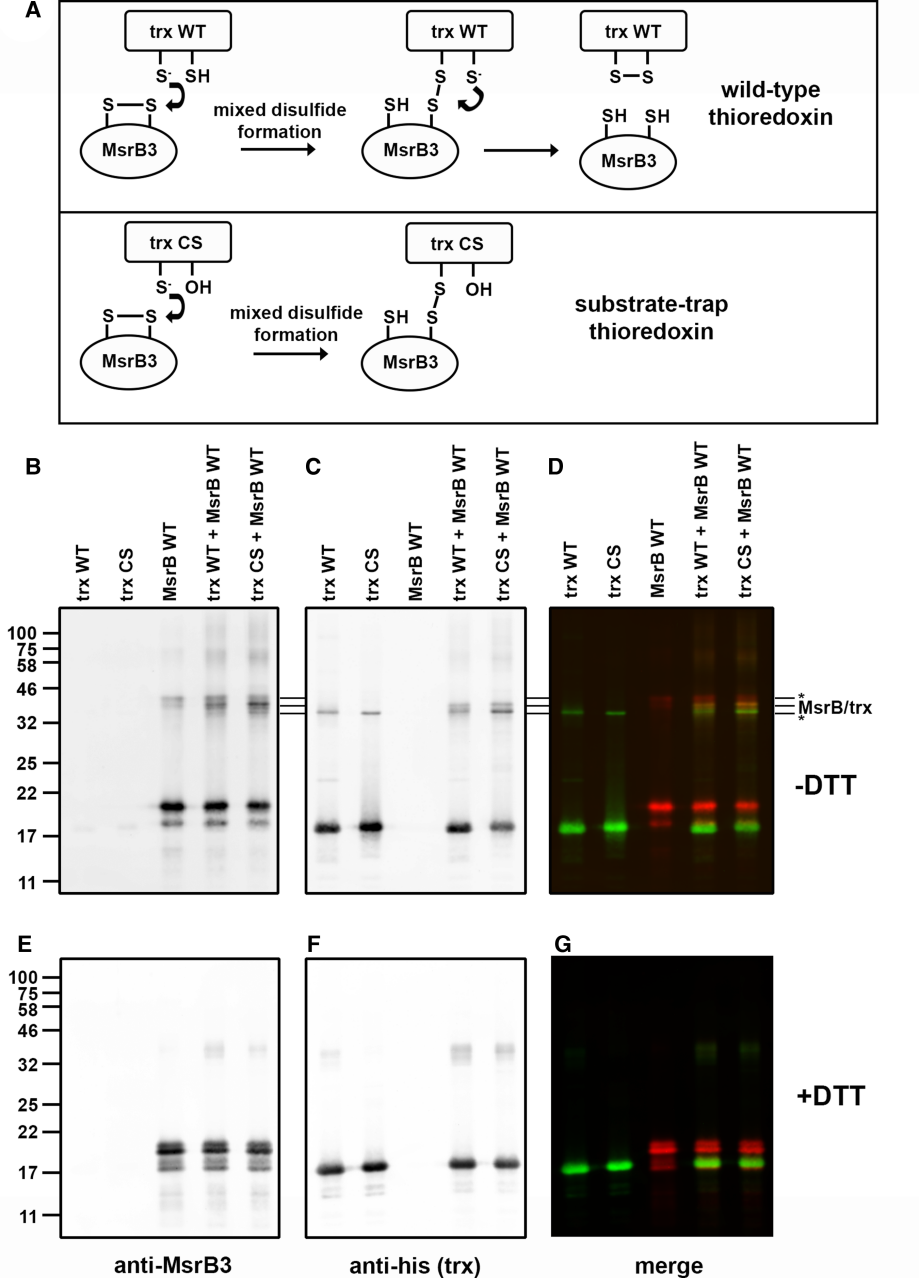
Formation of a mixed disulfide between MsrB3 and Trx. (**A**) Cartoon depicting the potential mechanism of reduction of a disulfide within MsrB3 by thioredoxin wild-type (WT) or a substrate-trapping mutant of thioredoxin (CS). In both cases, the first step of the reaction involves the formation of a mixed disulfide between the enzyme and the substrate. This is resolved with the WT protein resulting in reduction of the disulfide in MsrB3 and the formation of a disulfide in thioredoxin. As the second active site cysteine is mutated to a serine in the substrate-trapping mutant, the mixed disulfide intermediate persists allowing identification of a complex between the enzyme and the substrate. (**B**–**G**) WT (trx WT) or mutant (trx CS) thioredoxin was incubated with MsrB3A and methionine sulfoxide at 37°C for 1 h. The mixtures were then separated on an SDS–PAGE gel in the absence (**B–D**) or presence (**E–G**) of DTT and proteins identified by immunoblotting with anti-MsrB3 (red) (**B** and **E**) or anti-His (green) for thioredoxin (**C** and **F**). The blots were scanned with a Licor Odyssey at 700 and 800 nm. The image was split into two channels to identify protein bands containing MsrB3 (**B** and **E**) or thioredoxin (**C** and **F**). The image prior to channel splitting is also depicted (**D** and **G**) to illustrate co-migration of complexes containing both MsrB3 and thioredoxin (appear as yellow bands). The migration of MsrB3 and thioredoxin dimers (*) as well as the complex between MsrB3 and thioredoxin are indicated with three lines next to the gel images.

To determine the ability of thioredoxin to directly reduce the thiol groups within MsbB3, we made use of a substrate-trap mutant of thioredoxin. If the second cysteine within the CXXC catalytic motif of thioredoxin is mutated to serine, the resulting CXXS (CS) can initiate catalysis by the reduction of the disulfide in oxidised MsrB3 to form a mixed disulfide, but cannot resolve this mixed disulfide with the result being a stabilised covalent link between the enzyme and the substrate [26] (Figure 3A). When either wild-type or CS-thioredoxin was incubated with MsrB3 in the presence of methionine sulfoxide, mixed disulfides were formed that migrated at ∼40 kDa (Figure 3B–F). While mixed disulfides formed with wild-type thioredoxin, they were more prominent with the CS mutant. The mixed disulfide migrated between MsrB3 and thioredoxin dimers identified by western blotting with either antibodies to MsrB3 or the His-tagged thioredoxin (indicated by three lines in Figure 3B,C). The mixed disulfide was also verified by co-localisation of green and red fluorescence resulting in a yellow colour when the western blot was visualised before splitting the channels (Figure 3D). All mixed disulfides were lost when the samples were separated following reduction of disulfides with DTT (Figure 3E–G). These results demonstrate that thioredoxin can directly reduce oxidised MsrB3 to form a mixed disulfide.

**Figure 4.**
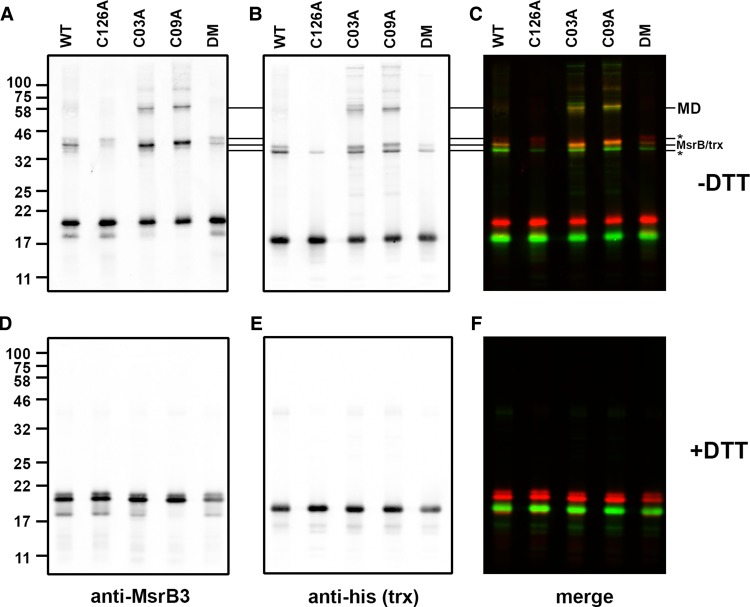
Formation of mixed disulfides between thioredoxin and MsrB3 cysteine mutants. (**A–F**) The substrate-trapping mutant of thioredoxin was incubated with WT and various cysteine mutants of MsrB3 as indicated and separated by SDS–PAGE either in the absence (**A–C**) or presence (**D–F**) of DTT. Immunoblotting, scanning and image presentation is as described in Figure 3.

Having established that thioredoxin can form a mixed disulfide to MsrB3, we then determined the consequence of mutating the active site or resolving cysteine residues, individually or together, on mixed disulfide formation (Figure 4). Mixed disulfides migrating at ∼40 kDa were observed for the wild-type, single resolving cysteine mutants (C03C and C09A) and to a much lesser extent, the double mutant. No mixed disulfides were observed to the active site cysteine mutant (C126A) despite the presence of both resolving cysteines. All mixed disulfides were lost when the samples were separated after reduction with DTT (Figure 4D–F). These results demonstrate that the active site cysteine is required for thioredoxin to form mixed disulfides and the presence of the resolving cysteines greatly enhances their formation.

We also observed additional higher molecular mass mixed disulfides between MsrB3 and thioredoxin, particularly with the resolving cysteine mutants (Figure 4A–C). These additional products are confirmed as mixed disulfides as they react with both antibodies to MsrB3 and thioredoxin. As we observe mixed disulfides between either C03, C09 or C126, these additional mixed disulfides are most likely to be MsrB3 with two thioredoxin molecules linked via C126 and either C03 or C09. Taken together, these results demonstrate that each resolving cysteine can form a disulfide with the active site thiol and that either of these disulfides can be a substrate for thioredoxin reduction.

### MsrB3 can act as a stereospecific oxidase generating the *R*-epimer

**Figure 5.**
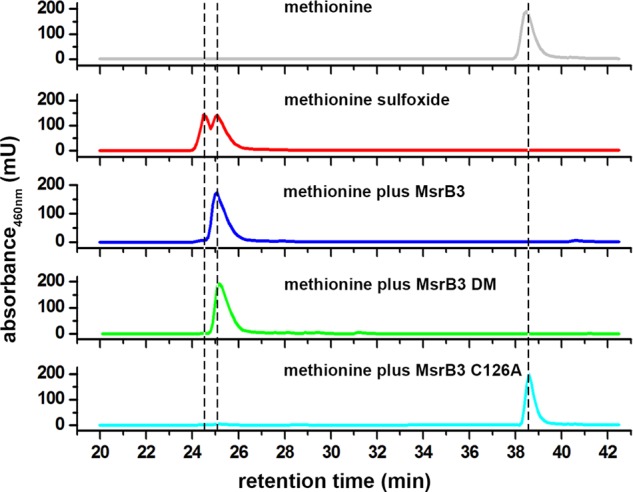
MsrB3 can act as a stereospecific oxidase. Msr3B was initially incubated with methionine sulfoxide in the absence of an electron donor to sulfenylate the active site thiol. After removal of methionine sulfoxide, the enzyme was incubated with dabsyl-methionine for 12 h at 37°C before RP-HPLC. The elution profiles of dabsyl-methionine (grey) and a mixture of dabsyl-methionine *S*- and *R-* sulfoxide (red) are shown to illustrate the retention times for these molecules. The elution profiles of reactants of dabsyl-methionine with WT MsrB3 (blue), MsrB3 DM (green) and MsrB3 C126A (cyan) are shown. No reaction occurs with MsrB3 C126A, but stoichiometric conversion of methionine into methionine *R*-sulfoxide occurs with both MsrB3 WT and DM.

The oxidation of methionine by chemicals such as hydrogen peroxide generates a mixture of the *S-* and *R*-epimers [6]. As MsrB3 is able to reduce only the *R*-epimer and is the only Msr within the ER, we evaluated its ability to act as an oxidase. We prepared the enzyme containing a sulfenylated thiol at its active site as described previously for MsrA [16] by incubating the enzyme with an excess of methionine sulfoxide. When this oxidised enzyme was incubated with stoichiometric amounts of methionine, we detected complete conversion to methionine *R*-sulfoxide (Figure 5). Both the wild type and the double mutant (DM) were able to catalyse this conversion, whereas the C126A active site mutant was inactive. These results demonstrate that, under the conditions used in this experiment, MsrB3 can act as a stereospecific oxidase and that this activity does not require the resolving cysteine residues or the presence of thioredoxin.

## Discussion

**Figure 6.**
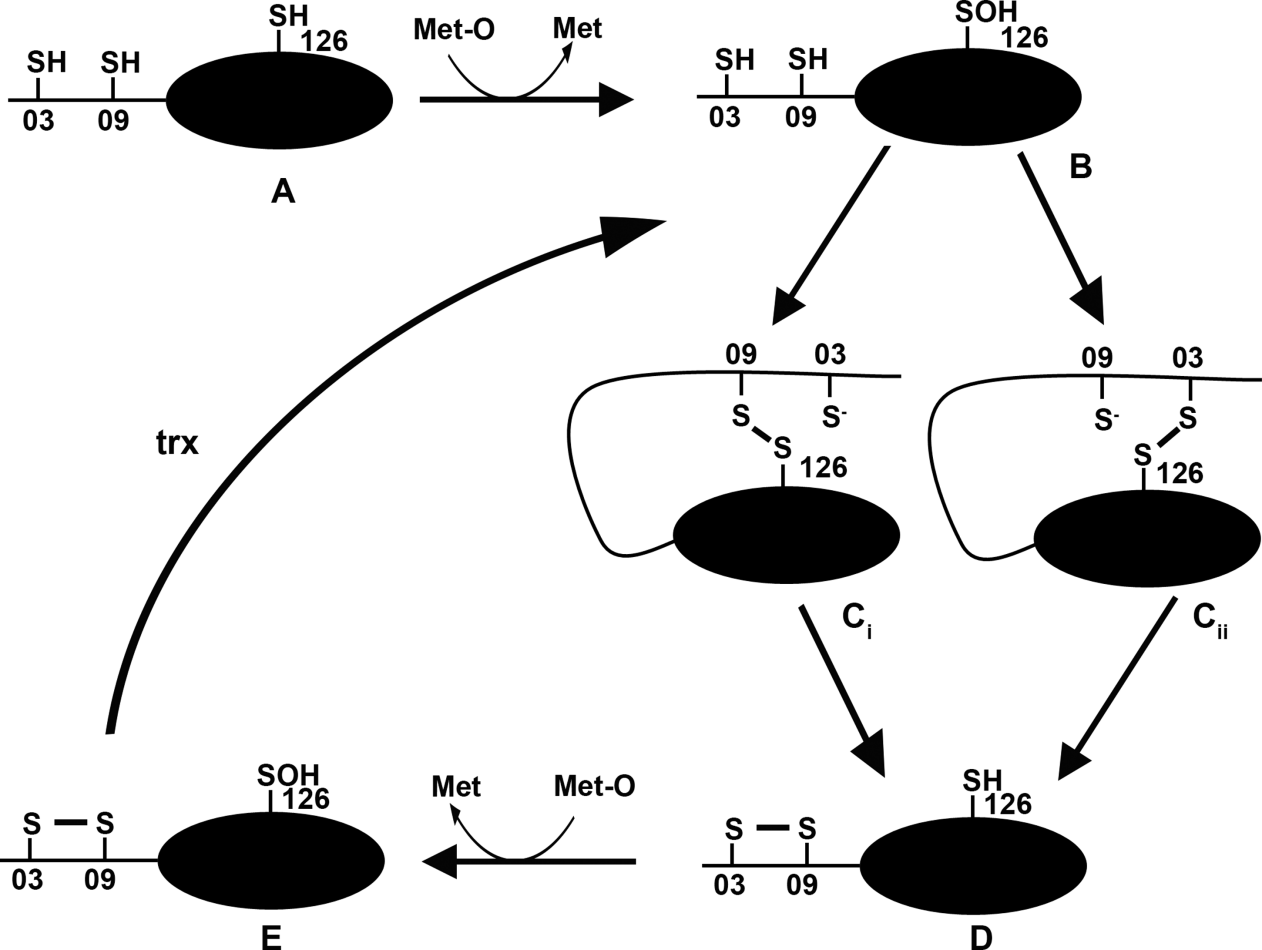
Overall reaction sequence for MsrB3. Cartoon depicting a potential reaction sequence for MsrB3 (adapted from ref. [17]). Fully reduced enzyme (**A**) reacts with methionine sulfoxide (Met-O) to form methionine and in the process the active site cysteine 126 becomes sulfenylated (**B**). This sulfenylated thiol can then be reduced by either cys03 or cys09 forming a transient disulfide between the active site and the resolving thiol (**C_i_** or **C_ii_**). After disulfide exchange, an internal disulfide is formed between cys 03 and 09 (**D**). A second molecule of methionine sulfoxide can then react with the active site cysteine to form E. The internal disulfide can then be reduced to regenerate (B).

Here, we have confirmed the specific reduction of methionine *R*-sulfoxide by MsrB3 and shown that the enzyme mechanism most likely involves a three-stage process as outlined (Figure 6). The essential difference from the previous mechanism suggested for this enzyme [11] is the presence of two resolving thiols that could work together or independently. Partial loss of activity when either of these two thiols were mutated suggests that either could function to directly reduce the active site sulfenylated thiol and that the presence of both thiols is required for full activity when thioredoxin is the electron donor. Removal of both thiols did not completely remove activity, suggesting that thioredoxin can directly reduce the active site thiol albeit inefficiently. This is supported by the ability to form mixed disulfides between the MsrB3 active site thiol and thioredoxin. However, the greater activity when the resolving thiols are present along with the more efficient formation of mixed disulfides to thioredoxin suggests that the preferred mechanism is via a resolving thiol.

As Msr3BA is localised to the ER lumen, thioredoxin will not be its physiological reductant. However, there are several thioredoxin domain-containing proteins in the ER including members of the protein disulfide isomerase (PDI) family. Strong candidates for PDIs which could act as a reductant include ERdj5 [27] or TXNDC11 [28], which each have relatively low reduction potentials. To complete the enzymatic cycle would require a thioredoxin reductase or an equivalent system within the ER lumen. No such enzyme has been identified to date, suggesting the requirement for an alternative source of reducing equivalents [29]. Possibilities include reduction by glutathione or the transfer of reducing equivalents from the thioredoxin pathway in the cytosol across the ER membrane. Such a pathway has been suggested recently to allow the reduction of non-native disulfides formed in some proteins during their normal folding pathway [30,31].

The ability of MsrB3 to act as a stereospecific oxidase forming methionine *R*-sulfoxide mirrors the ability of some MsrA enzymes to form methionine *S*-sulfoxide. While the oxidation reaction is much slower than reduction, it has been argued that under physiological conditions, the reaction may well be significant [14,17]. Such an oxidase activity is reliant upon the absence of rapid recycling of the sulfenylated active site thiol. Using a kinetic approach with MsrA, it was demonstrated that once the internal disulfide bond is formed between the resolving cysteines, then the rate of sulfenylation of the active site thiol is higher than that of reduction of the internal disulfide by thioredoxin [17]. This would allow the sulfenylated active site to catalyse the oxidation of its substrate. In regard to MsrB3, this would mean that once the disulfide between C03 and C09 had formed, C126 could become sulfenylated for sufficient time to allow oxidation. In addition, the absence of a robust thioredoxin reductase pathway in the ER may slow down the recycling pathway thereby prolonging the sulfenylated active site intermediate. Other ER enzyme, such as peroxiredoxin IV, requires reduction of a disulfide to maintain activity, and this reductive step has been demonstrated to be less efficient within the ER than the cytosol [32].

The physiological role of MsrB3A remains unknown due to the absence of data on the role and extent of methionine oxidation within the ER or the potential substrates for this enzyme. It is known that MsrB3A deficiency causes ER stress as well as an increased sensitivity of cancer cells to apoptosis [18,33]. These observations would suggest an important physiological role for methionine sulfoxide reduction in the ER to alleviate stress. The potential oxidase activity of MsrB3 suggests an alternative role in the regulation of protein function by reversible modification of methionine.

## Abbreviations

DM: double mutant
ER: endoplasmic reticulum
Msr: methionine sulfoxide reductases
PDI: protein disulfide isomerase
Trx: thioredoxin
trxR: thioredoxin reductase
TTBS: Tween/Tris-buffered saline
